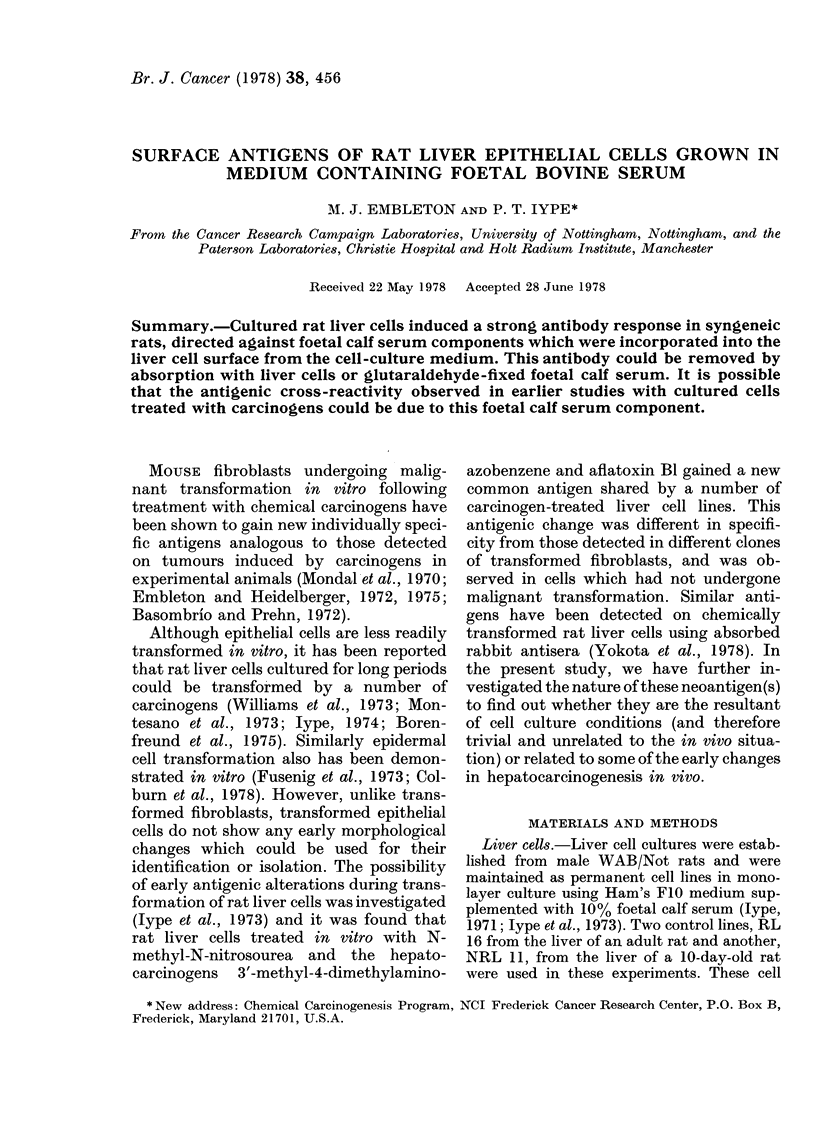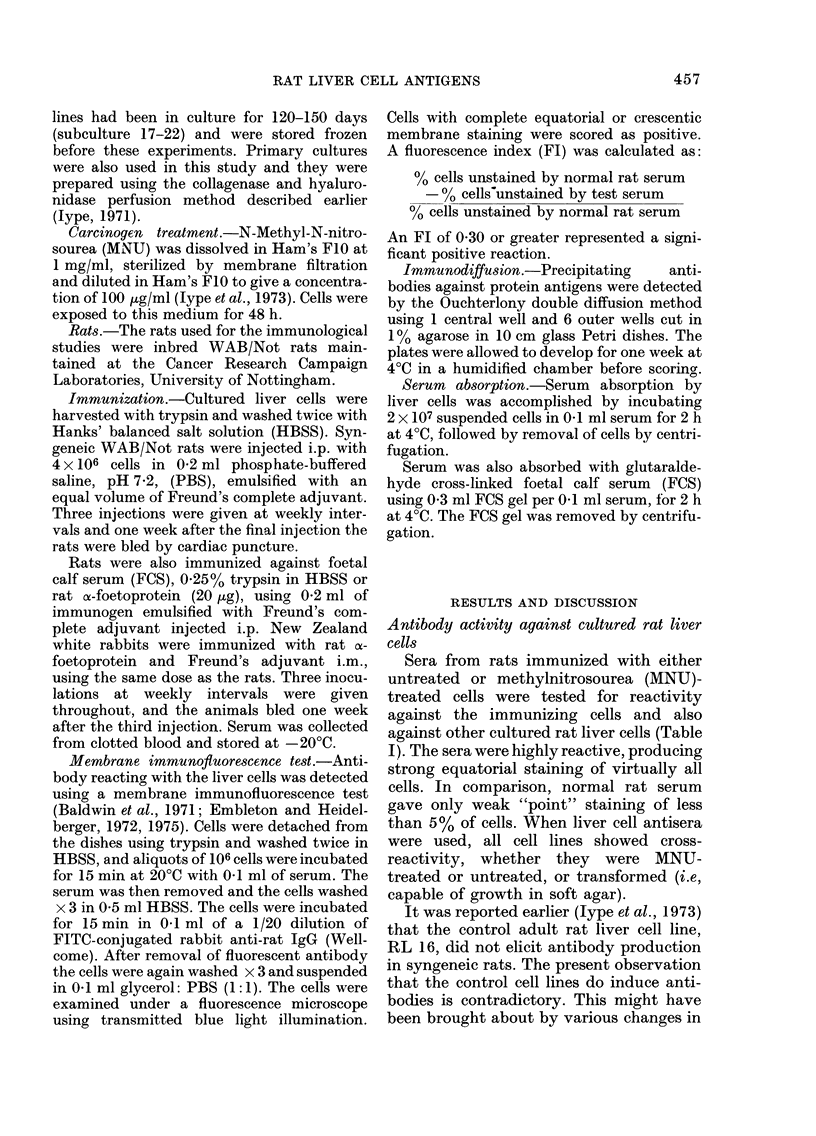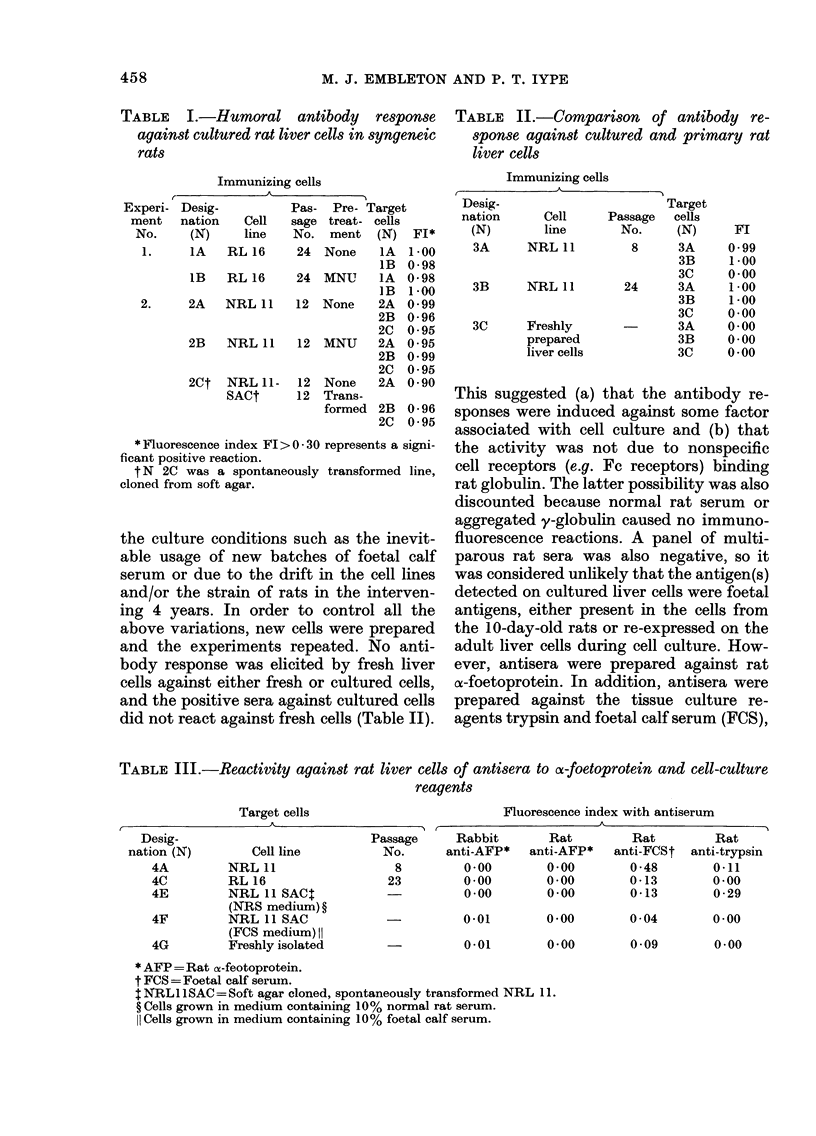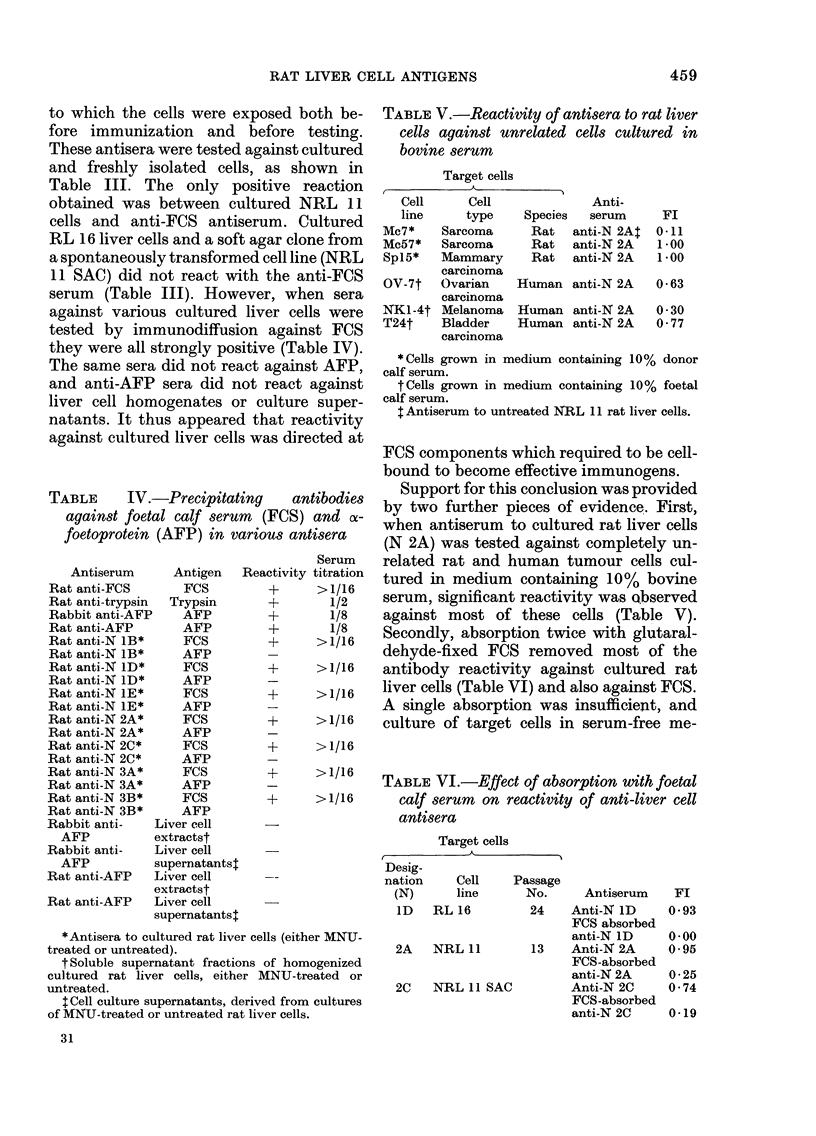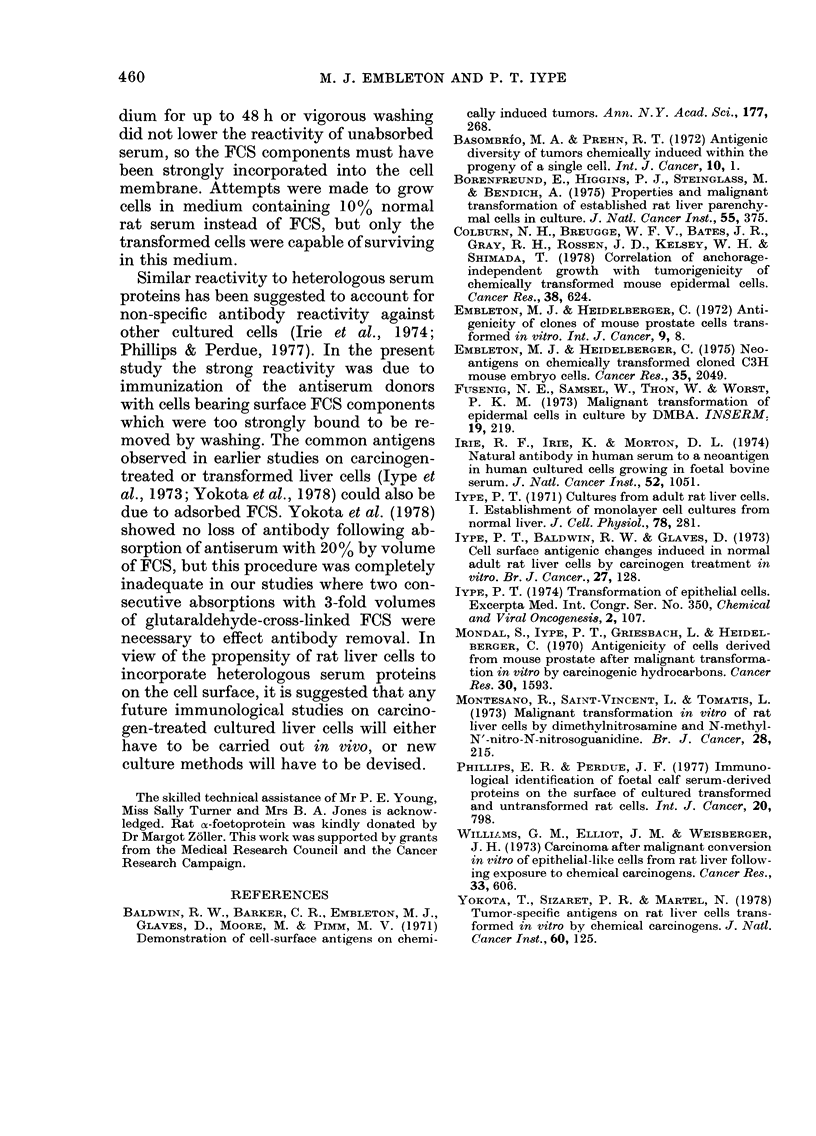# Surface antigens of rat liver epithelial cells grown in medium containing foetal bovine serum.

**DOI:** 10.1038/bjc.1978.229

**Published:** 1978-09

**Authors:** M. J. Embleton, P. T. Iype

## Abstract

Cultured rat liver cells induced a strong antibody response in syngeneic rats, directed against foetal calf serum components which were incorporated into the liver cell surface from the cell-culture medium. This antibody could be removed by absorption with liver cells or glutaraldehyde-fixed foetal calf serum. It is possible that the antigenic cross-reactivity observed in earlier studies with cultured cells treated with carcinogens could be due to this foetal calf serum component.


*Br. J. Cancer* (1978) **38**, 456

### SURFACE ANTIGENS OF RAT LIVER EPITHELIAL CELLS GROWN IN MEDIUM CONTAINING FOETAL BOVINE SERUM

M. J. EMBLETON AND P. T. IYPE*

*From the Cancer Research Campaign Laboratories, University of Nottingham, Nottingham, and the Paterson Laboratories, Christie Hospital and Holt Radium Institute, Manchester*

Received 22 May 1978 Accepted 28 June 1978

**Summary.—Cultured rat liver cells induced a strong antibody response in syngeneic rats, directed against foetal calf serum components which were incorporated into the liver cell surface from the cell-culture medium. This antibody could be removed by absorption with liver cells or glutaraldehyde-fixed foetal calf serum. It is possible that the antigenic cross-reactivity observed in earlier studies with cultured cells treated with carcinogens could be due to this foetal calf serum component.**

MOUSE fibroblasts undergoing malignant transformation *in vitro* following treatment with chemical carcinogens have been shown to gain new individually specific antigens analogous to those detected on tumours induced by carcinogens in experimental animals (Mondal *et al.*, 1970; Embleton and Heidelberger, 1972, 1975; Basombrío and Prehn, 1972).

Although epithelial cells are less readily transformed *in vitro*, it has been reported that rat liver cells cultured for long periods could be transformed by a number of carcinogens (Williams *et al.*, 1973; Montesano *et al.*, 1973; Iype, 1974; Borenfreund *et al.*, 1975). Similarly epidermal cell transformation also has been demonstrated *in vitro* (Fusenig *et al.*, 1973; Colburn *et al.*, 1978). However, unlike transformed fibroblasts, transformed epithelial cells do not show any early morphological changes which could be used for their identification or isolation. The possibility of early antigenic alterations during transformation of rat liver cells was investigated (Iype *et al.*, 1973) and it was found that rat liver cells treated *in vitro* with N-methyl-N-nitrosourea and the hepatocarcinogens 3′-methyl-4-dimethylaminoazobenzene and aflatoxin Bl gained a new common antigen shared by a number of carcinogen-treated liver cell lines. This antigenic change was different in specificity from those detected in different clones of transformed fibroblasts, and was observed in cells which had not undergone malignant transformation. Similar antigens have been detected on chemically transformed rat liver cells using absorbed rabbit antisera (Yokota *et al.*, 1978). In the present study, we have further investigated the nature of these neoantigen(s) to find out whether they are the resultant of cell culture conditions (and therefore trivial and unrelated to the *in vivo* situation) or related to some of the early changes in hepatocarcinogenesis *in vivo*.

### MATERIALS AND METHODS

*Liver cells.*—Liver cell cultures were established from male WAB/Not rats and were maintained as permanent cell lines in monolayer culture using Ham's F10 medium supplemented with 10% foetal calf serum (Iype, 1971; Iype *et al.*, 1973). Two control lines, RL 16 from the liver of an adult rat and another, NRL 11, from the liver of a 10-day-old rat were used in these experiments. These cell

* New address: Chemical Carcinogenesis Program, NCI Frederick Cancer Research Center, P.O. Box B, Frederick, Maryland 21701, U.S.A.

lines had been in culture for 120–150 days (subculture 17–22) and were stored frozen before these experiments. Primary cultures were also used in this study and they were prepared using the collagenase and hyaluronidase perfusion method described earlier (Iype, 1971).

*Carcinogen treatment.*—N-Methyl-N-nitrosourea (MNU) was dissolved in Ham's F10 at 1 mg/ml, sterilized by membrane filtration and diluted in Ham's F10 to give a concentration of 100 $\mu$g/ml (Iype *et al.*, 1973). Cells were exposed to this medium for 48 h.

*Rats.*—The rats used for the immunological studies were inbred WAB/Not rats maintained at the Cancer Research Campaign Laboratories, University of Nottingham.

*Immunization.*—Cultured liver cells were harvested with trypsin and washed twice with Hanks' balanced salt solution (HBSS). Syngeneic WAB/Not rats were injected i.p. with $4 \times 10^6$ cells in 0·2 ml phosphate-buffered saline, pH 7·2, (PBS), emulsified with an equal volume of Freund's complete adjuvant. Three injections were given at weekly intervals and one week after the final injection the rats were bled by cardiac puncture.

Rats were also immunized against foetal calf serum (FCS), 0·25% trypsin in HBSS or rat $\alpha$-foetoprotein (20 $\mu$g), using 0·2 ml of immunogen emulsified with Freund's complete adjuvant injected i.p. New Zealand white rabbits were immunized with rat $\alpha$-foetoprotein and Freund's adjuvant i.m., using the same dose as the rats. Three inoculations at weekly intervals were given throughout, and the animals bled one week after the third injection. Serum was collected from clotted blood and stored at −20°C.

*Membrane immunofluorescence test.*—Antibody reacting with the liver cells was detected using a membrane immunofluorescence test (Baldwin *et al.*, 1971; Embleton and Heidelberger, 1972, 1975). Cells were detached from the dishes using trypsin and washed twice in HBSS, and aliquots of $10^6$ cells were incubated for 15 min at 20°C with 0·1 ml of serum. The serum was then removed and the cells washed ×3 in 0·5 ml HBSS. The cells were incubated for 15 min in 0·1 ml of a 1/20 dilution of FITC-conjugated rabbit anti-rat IgG (Wellcome). After removal of fluorescent antibody the cells were again washed ×3 and suspended in 0·1 ml glycerol: PBS (1:1). The cells were examined under a fluorescence microscope using transmitted blue light illumination. Cells with complete equatorial or crescentic membrane staining were scored as positive. A fluorescence index (FI) was calculated as:

$$\frac{\%\text{ cells unstained by normal rat serum} - \%\text{ cells unstained by test serum}}{\%\text{ cells unstained by normal rat serum}}$$

An FI of 0·30 or greater represented a significant positive reaction.

*Immunodiffusion.*—Precipitating antibodies against protein antigens were detected by the Ouchterlony double diffusion method using 1 central well and 6 outer wells cut in 1% agarose in 10 cm glass Petri dishes. The plates were allowed to develop for one week at 4°C in a humidified chamber before scoring.

*Serum absorption.*—Serum absorption by liver cells was accomplished by incubating $2 \times 10^7$ suspended cells in 0·1 ml serum for 2 h at 4°C, followed by removal of cells by centrifugation.

Serum was also absorbed with glutaraldehyde cross-linked foetal calf serum (FCS) using 0·3 ml FCS gel per 0·1 ml serum, for 2 h at 4°C. The FCS gel was removed by centrifugation.

### RESULTS AND DISCUSSION

### *Antibody activity against cultured rat liver cells*

Sera from rats immunized with either untreated or methylnitrosourea (MNU)-treated cells were tested for reactivity against the immunizing cells and also against other cultured rat liver cells (Table I). The sera were highly reactive, producing strong equatorial staining of virtually all cells. In comparison, normal rat serum gave only weak "point" staining of less than 5% of cells. When liver cell antisera were used, all cell lines showed cross-reactivity, whether they were MNU-treated or untreated, or transformed (*i.e*, capable of growth in soft agar).

It was reported earlier (Iype *et al.*, 1973) that the control adult rat liver cell line, RL 16, did not elicit antibody production in syngeneic rats. The present observation that the control cell lines do induce antibodies is contradictory. This might have been brought about by various changes in

TABLE I.—*Humoral antibody response against cultured rat liver cells in syngeneic rats*

| | Immunizing cells | | | | | |
|---|---|---|---|---|---|---|
| Experiment No. | Designation (N) | Cell line | Passage No. | Pre-treatment | Target cells (N) | FI* |
| 1. | 1A | RL 16 | 24 | None | 1A | 1·00 |
| | | | | | 1B | 0·98 |
| | 1B | RL 16 | 24 | MNU | 1A | 0·98 |
| | | | | | 1B | 1·00 |
| 2. | 2A | NRL 11 | 12 | None | 2A | 0·99 |
| | | | | | 2B | 0·96 |
| | | | | | 2C | 0·95 |
| | 2B | NRL 11 | 12 | MNU | 2A | 0·95 |
| | | | | | 2B | 0·99 |
| | | | | | 2C | 0·95 |
| | 2C† | NRL 11-SAC† | 12 | None | 2A | 0·90 |
| | | | 12 | Transformed | 2B | 0·96 |
| | | | | | 2C | 0·95 |

*Fluorescence index FI>0·30 represents a significant positive reaction.
†N 2C was a spontaneously transformed line, cloned from soft agar.

the culture conditions such as the inevitable usage of new batches of foetal calf serum or due to the drift in the cell lines and/or the strain of rats in the intervening 4 years. In order to control all the above variations, new cells were prepared and the experiments repeated. No antibody response was elicited by fresh liver cells against either fresh or cultured cells, and the positive sera against cultured cells did not react against fresh cells (Table II).

TABLE II.—*Comparison of antibody response against cultured and primary rat liver cells*

| Immunizing cells | | | | |
|---|---|---|---|---|
| Designation (N) | Cell line | Passage No. | Target cells (N) | FI |
| 3A | NRL 11 | 8 | 3A | 0·99 |
| | | | 3B | 1·00 |
| | | | 3C | 0·00 |
| 3B | NRL 11 | 24 | 3A | 1·00 |
| | | | 3B | 1·00 |
| | | | 3C | 0·00 |
| 3C | Freshly prepared liver cells | — | 3A | 0·00 |
| | | | 3B | 0·00 |
| | | | 3C | 0·00 |

This suggested (a) that the antibody responses were induced against some factor associated with cell culture and (b) that the activity was not due to nonspecific cell receptors (*e.g.* Fc receptors) binding rat globulin. The latter possibility was also discounted because normal rat serum or aggregated $\gamma$-globulin caused no immunofluorescence reactions. A panel of multiparous rat sera was also negative, so it was considered unlikely that the antigen(s) detected on cultured liver cells were foetal antigens, either present in the cells from the 10-day-old rats or re-expressed on the adult liver cells during cell culture. However, antisera were prepared against rat $\alpha$-foetoprotein. In addition, antisera were prepared against the tissue culture reagents trypsin and foetal calf serum (FCS),

TABLE III.—*Reactivity against rat liver cells of antisera to $\alpha$-foetoprotein and cell-culture reagents*

| Target cells | | | Fluorescence index with antiserum | | | |
|---|---|---|---|---|---|---|
| Designation (N) | Cell line | Passage No. | Rabbit anti-AFP* | Rat anti-AFP* | Rat anti-FCS† | Rat anti-trypsin |
| 4A | NRL 11 | 8 | 0·00 | 0·00 | 0·48 | 0·11 |
| 4C | RL 16 | 23 | 0·00 | 0·00 | 0·13 | 0·00 |
| 4E | NRL 11 SAC‡ (NRS medium)§ | — | 0·00 | 0·00 | 0·13 | 0·29 |
| 4F | NRL 11 SAC (FCS medium)‖ | — | 0·01 | 0·00 | 0·04 | 0·00 |
| 4G | Freshly isolated | — | 0·01 | 0·00 | 0·09 | 0·00 |

*AFP = Rat $\alpha$-feotoprotein.
†FCS = Foetal calf serum.
‡NRL11SAC = Soft agar cloned, spontaneously transformed NRL 11.
§Cells grown in medium containing 10% normal rat serum.
‖Cells grown in medium containing 10% foetal calf serum.

to which the cells were exposed both before immunization and before testing. These antisera were tested against cultured and freshly isolated cells, as shown in Table III. The only positive reaction obtained was between cultured NRL 11 cells and anti-FCS antiserum. Cultured RL 16 liver cells and a soft agar clone from a spontaneously transformed cell line (NRL 11 SAC) did not react with the anti-FCS serum (Table III). However, when sera against various cultured liver cells were tested by immunodiffusion against FCS they were all strongly positive (Table IV). The same sera did not react against AFP, and anti-AFP sera did not react against liver cell homogenates or culture supernatants. It thus appeared that reactivity against cultured liver cells was directed at FCS components which required to be cell-bound to become effective immunogens.

TABLE IV.—*Precipitating antibodies against foetal calf serum* (FCS) *and α-foetoprotein* (AFP) *in various antisera*

| Antiserum | Antigen | Reactivity | Serum titration |
|---|---|---|---|
| Rat anti-FCS | FCS | + | >1/16 |
| Rat anti-trypsin | Trypsin | + | 1/2 |
| Rabbit anti-AFP | AFP | + | 1/8 |
| Rat anti-AFP | AFP | + | 1/8 |
| Rat anti-N 1B* | FCS | + | >1/16 |
| Rat anti-N 1B* | AFP | − | |
| Rat anti-N 1D* | FCS | + | >1/16 |
| Rat anti-N 1D* | AFP | − | |
| Rat anti-N 1E* | FCS | + | >1/16 |
| Rat anti-N 1E* | AFP | − | |
| Rat anti-N 2A* | FCS | + | >1/16 |
| Rat anti-N 2A* | AFP | − | |
| Rat anti-N 2C* | FCS | + | >1/16 |
| Rat anti-N 2C* | AFP | − | |
| Rat anti-N 3A* | FCS | + | >1/16 |
| Rat anti-N 3A* | AFP | − | |
| Rat anti-N 3B* | FCS | + | >1/16 |
| Rat anti-N 3B* | AFP | | |
| Rabbit anti-AFP | Liver cell extracts† | — | |
| Rabbit anti-AFP | Liver cell supernatants‡ | — | |
| Rat anti-AFP | Liver cell extracts† | — | |
| Rat anti-AFP | Liver cell supernatants‡ | — | |

*Antisera to cultured rat liver cells (either MNU-treated or untreated).

†Soluble supernatant fractions of homogenized cultured rat liver cells, either MNU-treated or untreated.

‡Cell culture supernatants, derived from cultures of MNU-treated or untreated rat liver cells.

TABLE V.—*Reactivity of antisera to rat liver cells against unrelated cells cultured in bovine serum*

| Target cells | | | | |
|---|---|---|---|---|
| Cell line | Cell type | Species | Anti-serum | FI |
| Mc7* | Sarcoma | Rat | anti-N 2A‡ | 0·11 |
| Mc57* | Sarcoma | Rat | anti-N 2A | 1·00 |
| Sp15* | Mammary carcinoma | Rat | anti-N 2A | 1·00 |
| OV-7† | Ovarian carcinoma | Human | anti-N 2A | 0·63 |
| NK1-4† | Melanoma | Human | anti-N 2A | 0·30 |
| T24† | Bladder carcinoma | Human | anti-N 2A | 0·77 |

*Cells grown in medium containing 10% donor calf serum.

†Cells grown in medium containing 10% foetal calf serum.

‡Antiserum to untreated NRL 11 rat liver cells.

Support for this conclusion was provided by two further pieces of evidence. First, when antiserum to cultured rat liver cells (N 2A) was tested against completely unrelated rat and human tumour cells cultured in medium containing 10% bovine serum, significant reactivity was observed against most of these cells (Table V). Secondly, absorption twice with glutaraldehyde-fixed FCS removed most of the antibody reactivity against cultured rat liver cells (Table VI) and also against FCS. A single absorption was insufficient, and culture of target cells in serum-free me-

TABLE VI.—*Effect of absorption with foetal calf serum on reactivity of anti-liver cell antisera*

| Target cells | | | | |
|---|---|---|---|---|
| Designation (N) | Cell line | Passage No. | Antiserum | FI |
| 1D | RL 16 | 24 | Anti-N 1D | 0·93 |
| | | | FCS absorbed anti-N 1D | 0·00 |
| 2A | NRL 11 | 13 | Anti-N 2A | 0·95 |
| | | | FCS-absorbed anti-N 2A | 0·25 |
| 2C | NRL 11 SAC | | Anti-N 2C | 0·74 |
| | | | FCS-absorbed anti-N 2C | 0·19 |

dium for up to 48 h or vigorous washing did not lower the reactivity of unabsorbed serum, so the FCS components must have been strongly incorporated into the cell membrane. Attempts were made to grow cells in medium containing 10% normal rat serum instead of FCS, but only the transformed cells were capable of surviving in this medium.

Similar reactivity to heterologous serum proteins has been suggested to account for non-specific antibody reactivity against other cultured cells (Irie *et al.*, 1974; Phillips & Perdue, 1977). In the present study the strong reactivity was due to immunization of the antiserum donors with cells bearing surface FCS components which were too strongly bound to be removed by washing. The common antigens observed in earlier studies on carcinogen-treated or transformed liver cells (Iype *et al.*, 1973; Yokota *et al.*, 1978) could also be due to adsorbed FCS. Yokota *et al.* (1978) showed no loss of antibody following absorption of antiserum with 20% by volume of FCS, but this procedure was completely inadequate in our studies where two consecutive absorptions with 3-fold volumes of glutaraldehyde-cross-linked FCS were necessary to effect antibody removal. In view of the propensity of rat liver cells to incorporate heterologous serum proteins on the cell surface, it is suggested that any future immunological studies on carcinogen-treated cultured liver cells will either have to be carried out *in vivo*, or new culture methods will have to be devised.

The skilled technical assistance of Mr P. E. Young, Miss Sally Turner and Mrs B. A. Jones is acknowledged. Rat α-foetoprotein was kindly donated by Dr Margot Zöller. This work was supported by grants from the Medical Research Council and the Cancer Research Campaign.

### REFERENCES

Baldwin, R. W., Barker, C. R., Embleton, M. J., Glaves, D., Moore, M. & Pimm, M. V. (1971) Demonstration of cell-surface antigens on chemically induced tumors. *Ann. N.Y. Acad. Sci.*, **177**, 268.

Basombrío, M. A. & Prehn, R. T. (1972) Antigenic diversity of tumors chemically induced within the progeny of a single cell. *Int. J. Cancer*, **10**, 1.

Borenfreund, E., Higgins, P. J., Steinglass, M. & Bendich, A. (1975) Properties and malignant transformation of established rat liver parenchymal cells in culture. *J. Natl. Cancer Inst.*, **55**, 375.

Colburn, N. H., Breugge, W. F. V., Bates, J. R., Gray, R. H., Rossen, J. D., Kelsey, W. H. & Shimada, T. (1978) Correlation of anchorage-independent growth with tumorigenicity of chemically transformed mouse epidermal cells. *Cancer Res.*, **38**, 624.

Embleton, M. J. & Heidelberger, C. (1972) Antigenicity of clones of mouse prostate cells transformed *in vitro*. *Int. J. Cancer*, **9**, 8.

Embleton, M. J. & Heidelberger, C. (1975) Neoantigens on chemically transformed cloned C3H mouse embryo cells. *Cancer Res.*, **35**, 2049.

Fusenig, N. E., Samsel, W., Thon, W. & Worst, P. K. M. (1973) Malignant transformation of epidermal cells in culture by DMBA. *INSERM*, **19**, 219.

Irie, R. F., Irie, K. & Morton, D. L. (1974) Natural antibody in human serum to a neoantigen in human cultured cells growing in foetal bovine serum. *J. Natl. Cancer Inst.*, **52**, 1051.

Iype, P. T. (1971) Cultures from adult rat liver cells. I. Establishment of monolayer cell cultures from normal liver. *J. Cell. Physiol.*, **78**, 281.

Iype, P. T., Baldwin, R. W. & Glaves, D. (1973) Cell surface antigenic changes induced in normal adult rat liver cells by carcinogen treatment *in vitro*. *Br. J. Cancer.*, **27**, 128.

Iype, P. T. (1974) Transformation of epithelial cells. Excerpta Med. Int. Congr. Ser. No. 350, *Chemical and Viral Oncogenesis*, **2**, 107.

Mondal, S., Iype, P. T., Griesbach, L. & Heidelberger, C. (1970) Antigenicity of cells derived from mouse prostate after malignant transformation *in vitro* by carcinogenic hydrocarbons. *Cancer Res.* **30**, 1593.

Montesano, R., Saint-Vincent, L. & Tomatis, L. (1973) Malignant transformation *in vitro* of rat liver cells by dimethylnitrosamine and N-methyl-N′-nitro-N-nitrosoguanidine. *Br. J. Cancer*, **28**, 215.

Phillips, E. R. & Perdue, J. F. (1977) Immunological identification of foetal calf serum-derived proteins on the surface of cultured transformed and untransformed rat cells. *Int. J. Cancer*, **20**, 798.

Williams, G. M., Elliot, J. M. & Weisberger, J. H. (1973) Carcinoma after malignant conversion *in vitro* of epithelial-like cells from rat liver following exposure to chemical carcinogens. *Cancer Res.*, **33**, 606.

Yokota, T., Sizaret, P. R. & Martel, N. (1978) Tumor-specific antigens on rat liver cells transformed *in vitro* by chemical carcinogens. *J. Natl. Cancer Inst.*, **60**, 125.